# Supplementary material for: Genetic and Metabolic Determinants of Atrial Fibrillation in a General Population Sample: The CHRIS Study
Source: Biomolecules. 2021 Nov 9;11(11):1663. doi: 10.3390/biom11111663 (PMC8615508; doi:10.3390/biom11111663)
Supplement: Supplementary file 1 [file biomolecules-11-01663-s001.zip › SUPP_Files/Table S1.docx]

**Table S1:** AF common risk factors data collection.

| **TRAIT** | **DATA COLLECTION PROTOCOL** |
| --- | --- |
| Age at participation, years | Numeric; Age at participation (rounded to the nearest full year)  (Date of participation - Birth date)/ 365.25 |
| Sex | 1 Male; 2 Female |
| Body Mass Index (BMI) | Numeric; Measured at study center visit.  Using OMRON Body Composition Monitor BF508 |
| Systolic Blood Pressure | Numeric (mm/Hg); Measured at study center visit.  Until 03/06/2015: Using Omron M10-IT  After 05/06/2015: Using CNAP Monitor 500 |
| Diastolic Blood Pressure | Numeric (mm/Hg); Measured at study center visit.  Until 03/06/2015: Using Omron M10-IT  After 05/06/2015: Using CNAP Monitor 500 |
| C-Reactive Protein | Numeric (mg/dL); Measured from serum obtained at study center visit.  Until 07/04/2014: Using ROCHE MODULAR PPE MODULAR PE; Cobas Tina-quant C-Reactive Protein Gen.3 kit  After 08/04/2014: Using ABBOTT DIAGNOSTIC ARCHITECT; CRP VARIO kit |
| Creatinine | Numeric (mg/dL); Measured from serum obtained at study center visit.  Until 07/04/2014: Using ROCHE MODULAR PPE MODULAR PE; Cobas Creatinine Jaffé Methode kit  After 08/04/2014: Using ABBOTT DIAGNOSTIC ARCHITECT; Creatinine kit |
| eGFR | Numeric (mL/min/1.73 m2); Measured from serum obtained at study center visit.  Derived from serum creatinine value using function “CKDEpi.creat” of the R package "nephro" |
| INTERVIEW: Smoking Habit | Factor: 1 Never smoker; 2 Past smoker; 3 Current smoker who reduced; 4 Current smoker who did not reduce; 5 Current smoker with unknown reduction status.  Ascertained in interview at study center visit from the following questions:   1. Participant was asked: "Have you ever smoked for as long as a year?" (Response: Yes*/No)   * Yes means at least 20 packs of cigarettes or 12 oz (360 grams) of tobacco in a lifetime, or at least one cigarette per day or one cigar a week for one year.   1. Participant was asked: "Do you currently smoke cigarettes?" (Response: Yes/No) 2. Participant was asked: "Have you stopped or cut down smoking" (Response: Yes/No) |
| INTERVIEW: Has a doctor ever said that you have high blood pressure or hypertension? | Ascertained in interview at study center visit from one of the following questions:   1. Participant was asked: "Has a doctor ever said that you have high blood pressure or hypertension?" (Response: Yes/No) 2. Participant was asked: "Have you ever been told that you have elevated or high blood pressure?" (Response: Yes/No) |
| INTERVIEW: Do you have diabetes mellitus? | Ascertained in interview at study center visit from the following question:  Participant was asked: "Do you have diabetes mellitus?" (Response: Yes/No) |
| INTERVIEW: Has a doctor ever told you that you have a heart failure? | Ascertained in interview at study center visit from the following question:  Participant was asked: "Has a doctor ever told you that you have a heart failure?" (Response: Yes/No) |
| INTERVIEW: Have you ever been told by a doctor that you had a stroke? | Ascertained in interview at study center visit from the following question:  Participant was asked: "Have you ever been told by a doctor that you had a stroke?" (Response: Yes/No) |
| INTERVIEW: Have you ever been told by a doctor that you had a myocardial infarction? | Ascertained in interview at study center visit from the following question:  Participant was asked: "Have you ever been told by a doctor that you had a myocardial infarction?” (Response: Yes/No) |
