# Supplementary material for: Genetic and Metabolic Determinants of Atrial Fibrillation in a General Population Sample: The CHRIS Study
Source: Biomolecules. 2021 Nov 9;11(11):1663. doi: 10.3390/biom11111663 (PMC8615508; doi:10.3390/biom11111663)
Supplement: Supplementary file 1 [file biomolecules-11-01663-s001.zip › SUPP_Files/Table S2.docx]

**Table S2**: Active compounds used to treat AF and their dosage. Compounds found in the current dataset and rated by an experienced cardiologist as a possible therapy for AF. ATC= Anatomical Therapeutic Chemical classification. All drugs are tablets apart from flecainide intravenous (IV).

| **ATC** | **Active compound** | **Dosage** | **Taking frequency** | **Taking mode** |
| --- | --- | --- | --- | --- |
| C01AA05 | Digoxin | 0.125 mg  0.2 mg | daily | regularly |
| C01AA08 | Metildigoxin | 0.1 mg | daily | regularly |
| C01BC03 | Propafenone | 450 mg | daily | regularly |
| C01BC04 | Flecainide | 50 mg  100 mg  150 mg  200 mg | daily | regularly |
| C01BC04 | Flecainide IV | 150 mg/15 ml | daily | regularly |
| C07AA07 | Sotalol | 120 mg  160 mg  200 mg | daily | regularly |
