# Supplementary material for: Genetic and Metabolic Determinants of Atrial Fibrillation in a General Population Sample: The CHRIS Study
Source: Biomolecules. 2021 Nov 9;11(11):1663. doi: 10.3390/biom11111663 (PMC8615508; doi:10.3390/biom11111663)
Supplement: Supplementary file 1 [file biomolecules-11-01663-s001.zip › SUPP_Files/Table S3.docx]

| **Chr** | **POS (GRCh37)** | **REF/ALT** | **rsID** | **MAF** | **MAC** | ***p*-value** | **R2** | **ALFA**  **SAMP#** | **ALFA MAF** |
| --- | --- | --- | --- | --- | --- | --- | --- | --- | --- |
| 1 | 164324085 | C/A | rs750729995 | 0.00055 | 11 | 5.3x10^-8^ | 0.59 | 9798 | 0.0000 |
| 1 | 165216674 | A/G | rs747358876 | 0.00042 | 9 | 5.3x10^-8^ | 0.48 | 9824 | 0.0003 |
| 1 | 164250567 | G/A | rs535411007 | 0.00076 | 15 | 7.3x10^-8^ | 0.65 | 9824 | 0.0011 |
| 13 | 101089416 | T/A | rs536973340 | 0.00046 | 13 | 1.4x10^-7^ | 0.72 | 9824 | 0.0003 |
| 8 | 8883613 | C/T | rs189051215 | 0.00635 | 137 | 2.8x10^-7^ | 0.78 | 14286 | 0.00476 |
| 13 | 101395796 | T/C | rs535535747 | 0.00047 | 12 | 3.0x10^-7^ | 0.83 | 9824 | 0.0005 |
| 17 | 17103013 | T/C | rs12939857 | 0.10852 | 2201 | 5.5x10^-7^ | 0.80 | 14286 | 0.13489 |
| 2 | 20776435 | C/T | rs182836018 | 0.00088 | 14 | 7.1x10^-7^ | 0.54 | 14286 | 0.00112 |
| 4 | 103113814 | G/A | rs77616117 | 0.00046 | 8 | 8.5x10^-7^ | 0.55 | 14286 | 0.00273 |

**Table S3**: AF-associated SNPs with GWAS *p*-value between 1 × 10^-6^ and 5 × 10^-8^

ALFA allele frequency data (for European populations) were retrieved from the NCBI SNP database 26-October-2021 (ALFA Project Release Version: 20201027095038). SAMP#: Sample count.
