# Supplementary material for: Genetic and Metabolic Determinants of Atrial Fibrillation in a General Population Sample: The CHRIS Study
Source: Biomolecules. 2021 Nov 9;11(11):1663. doi: 10.3390/biom11111663 (PMC8615508; doi:10.3390/biom11111663)
Supplement: Supplementary file 1 [file biomolecules-11-01663-s001.zip › SUPP_Files/Table S4.docx]

**Table S4:** Demographic characteristics and common risk factors in carriers and non-carriers AF cases.

|  | **Carriers (n=6)** | | | **Non Carriers (n=104)** | | |  |
| --- | --- | --- | --- | --- | --- | --- | --- |
|  | missings, n, (%) | mean | Min/Max | missings, n, (%) | mean | Min/Max | p-value |
|  |  | (SD) |  |  | (SD) |  |  |
| **Age at participation – years** | 0 | 51.67  (22.52) | 24/79 | 0 | 67.37  (19.25) | 18/93 | 0.0568 |
| **Sex, n (%)** | | | | | | | |
| male | 3 (50%) | | | 64 (61.5%) | | | 0.677 |
| female | 3 (50%) | | | 40 (38.5%) | | |  |
| **Body Mass Index – kg/m2** | 0 | 23.38  (3.15) | 19.1/28.3 | 5 (4.8%) | 28.1  (4.64) | 18.3/40.2 | 0.0159 |
| **Systolic BP,**  **mean of 3 measurements – mm/Hg** | 1 (16.7%) | 128.20 (26.25) | 96/166 | 4 (3.8%) | 131.61 (18.20) | 92/175 | 0.6896 |
| **Diastolic BP,**  **mean of 3 measurements – mm/Hg** | 1 (16.7%) | 84.40  (11.87) | 68/100 | 4 (3.8%) | 80.97 (11.45) | 59/122 | 0.5152 |
| **Cholesterol total – mg/dL** | 0 | 199.17 (40.87) | 141/240 | 0 | 196.86 (38.88) | 95/287 | 0.8879 |
| **Cholesterol**  **High Density Lipoprotein (HDL) – mg/dL** | 0 | 69.00  (12.81) | 55/92 | 0 | 55.90 (15.23) | 27/92 | 0.0416 |
| **Cholesterol**  **Low Density Lipoprotein (LDL) – mg/dL** | 0 | 119.17 (37.11) | 78/169 | 0 | 120.19 (35.82) | 31/220 | 0.9458 |
| **C-Reactive Protein (CRP) - mg/dL** | 0 | 0.50 (0.42) | 0.05/1.12 | 0 | 0.41 (0.49) | 0.02/2.85 | 0.6608 |
| **Creatinine (urine) - mg/dL** | 0 | 98.10  (55.88) | 30.20/188 | 0 | 103.27 (57.33) | 13.30/333 | 0.8302 |
| **eGFR**   **- mL/min/1.73 m²** | 0 | 77.13 (9.01) | 62.65/85.04 | 0 | 73.02 (22.49) | 26.06/134.25 | 0.6576 |
| **Smoking habit, n (%)** | | | | | | | |
| never | 2 (33.3%) | | | 58 (55.8%) | | | 0.327 |
| past | 3 (50%) | | | 36 (34.6%) | | |  |
| current | 1 (16.7%) | | | 9 (8.7%) | | |  |
| missing | 0 | | | 1 (0.9%) | | |  |
| **Has a doctor ever said that you have high blood pressure or hypertension? n (%)** | | | | | | | |
| yes | 3 (50%) | | | 53 (51%) | | | 1 |
| no | 3 (50%) | | | 50 (48.1%) | | |  |
| missing | 0 | | | 1 (0.9%) | | |  |
| **Do you have diabetes mellitus? n (%)** | | | | | | | |
| yes | 0 | | | 11 (10.6%) | | | 1 |
| no | 6 (100%) | | | 92 (88.5%) | | |  |
| missing | 0 | | | 1 (0.9%) | | |  |
| **Has a doctor ever told you that you have a heart failure? n (%)** | | | | | | | |
| yes | 1 (16.7%) | | | 16 (15.4%) | | | 1 |
| no | 5 (83.3%) | | | 81 (77.9%) | | |  |
| missing | 0 | | | 7 (6.7%) | | |  |
| **Have you ever been told by a doctor that you had a stroke? n (%)** | | | | | | | |
| yes | 0 | | | 13 (12.5%) | | | 1 |
| no | 6 (100%) | | | 90 (86.5%) | | |  |
| missing | 0 | | | 1 (0.9%) | | |  |
| **Have you ever been told by a doctor that you had a myocardial infarction? n (%)** | | | | | | | |
| yes | 0 | | | 9 (8.6%) | | | 1 |
| no | 6 (100%) | | | 92 (88.5%) | | |  |
| missing | 0 | | | 3 (2.9%) | | |  |

n= number; SD= standard deviation; BP= blood pressure; eGFR= estimated glomerular filtration rate; T test was used for continuous variables and Fisher's exact test for categorical variables.
