# Supplementary figures and images for: Genetic and Metabolic Determinants of Atrial Fibrillation in a General Population Sample: The CHRIS Study
Source: Biomolecules. 2021 Nov 9;11(11):1663. doi: 10.3390/biom11111663 (PMC8615508; doi:10.3390/biom11111663)

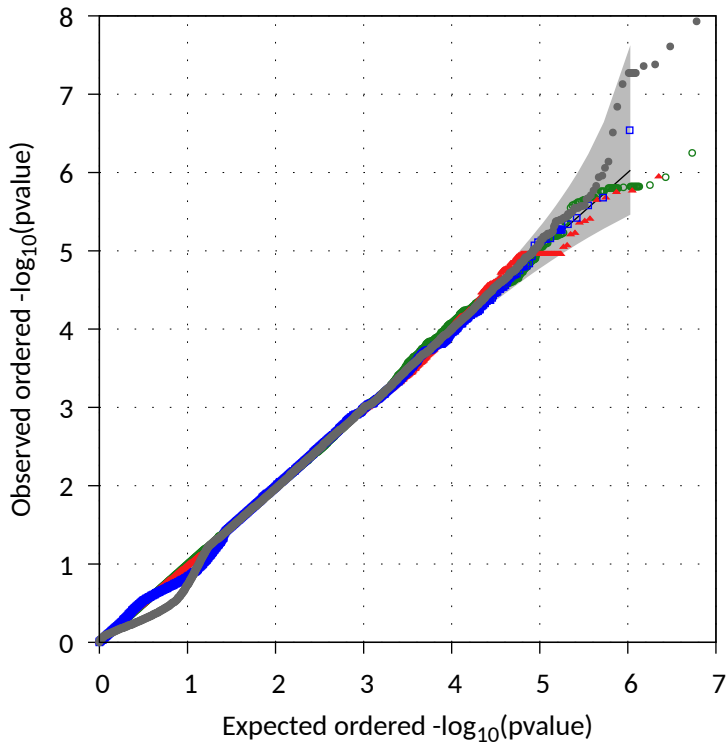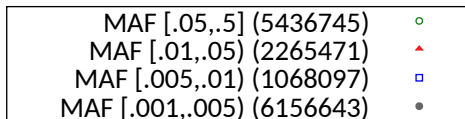

Supplement: Supplementary file 1 [file biomolecules-11-01663-s001.zip › SUPP_Files/Figure S1.pdf]

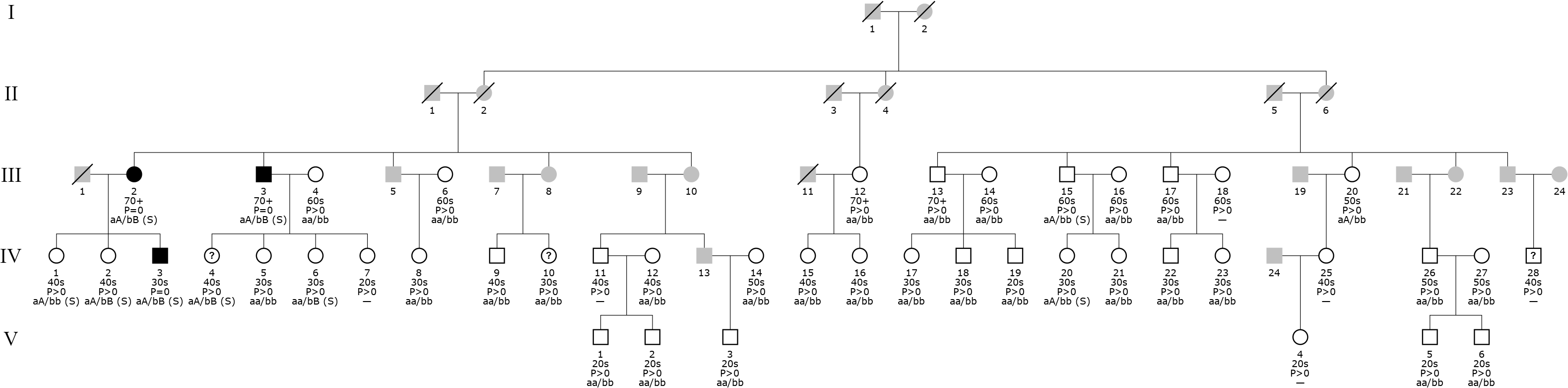

Supplement: Supplementary file 1 [file biomolecules-11-01663-s001.zip › SUPP_Files/Figure S2.png]
